# Vascularised fibula graft for tumours

**DOI:** 10.1186/1753-6561-9-S3-A99

**Published:** 2015-05-19

**Authors:** Emmanuel P Estrella

**Affiliations:** 1 University of the Philippines, Manila, Philippines

## 

The reconstruction of long bone defects after tumor resection has been a challenge to the reconstructive surgeon. Increased survival of patients with aggressive or malignant tumors of the extremities has made microsurgical reconstruction more relevant in improving their quality of life. In order to avoid amputation, these long defects are often reconstructed with vascularized autogenous fibular grafts. Vascularized fibular grafts have been reported to have a high union rate and good functional outcome. Union rates for vascularized fibular grafts range from 68-100% in individual case series. However, complications are also frequent. The reported complication rates range from 10-60%, with infection and non-union as the most frequent complications. Revision surgery in the form of additional bone grafting is often needed as well as revision of fixation of the graft.

The devices used for fibular autograft fixation include plate and screws, intramedullary nails, circular external fixator, and a combination of K-wires and screws with casting. Rigid fixation is necessary to achieve early adjacent joint motion and prevent graft non-union. Aside from bone union, functional assessment is important in the evaluation of patients treated with microsurgical reconstruction using the fibular graft. The most frequently used functional tool was the modified Enneking MSTS score (Musculoskeletal Tumor Society Score).

A retrospective review of patients diagnosed with benign aggressive or malignant extremity tumors with post-oncologic long bone extremity defects reconstructed with vascularized fibula grafts was undertaken from 1993 to 2008 to determine clinical outcome, bone union and functional outcome. A total of 25 patients were able to fulfill the inclusion criteria and were included in the review. Of the 25 patients, eight had benign-aggressive tumors while 17 had malignant tumors. Results showed a union rate of 84% (21/25). Revision surgery was done on seven patients (28%) to achieve union, all of them in the upper extremity. Three patients had infections (12%), and only 2 grafts had fractured (8%). The average length of the fibular graft was 18.22 ± 3.5 cm. Final union time for the grafts to unite was 10.4 ± 4.1 months. The average functional score using the Musculoskeletal Tumor Score in 20 patients was 83.4% (SD, 10.4). The average follow-up was 41 (SD, 32) months. On the latest follow-up, of the 14 patients with malignant bone lesions, two died of the disease and one died of other causes, the rest of the patients with malignant lesions are alive with no evidence of disease.

In summary, the use of vascularized fibula autografts for long bone defects after tumor resection represents a valid option of reconstruction. Union rates are high and complication rates are manageable.

Among the factors investigated, only graft union was significantly associated with the MSTS score. Patients whose graft united tend to have an MSTS score of 13.8 percentage points higher than those patients who had non-unions.

